# Multidimensional fragmentomic profiling of cell-free DNA released from patient-derived organoids

**DOI:** 10.1186/s40246-023-00533-0

**Published:** 2023-10-28

**Authors:** Jaeryuk Kim, Seung-Pyo Hong, Seyoon Lee, Woochan Lee, Dakyung Lee, Rokhyun Kim, Young Jun Park, Sungji Moon, Kyunghyuk Park, Bukyoung Cha, Jong-Il Kim

**Affiliations:** 1 Genomic Medicine Institute, Medical Research Center, Seoul National University, Seoul, Republic of Korea; 2 Department of Biomedical Sciences, Seoul National University Graduate School, Seoul, Republic of Korea; 3 Department of Translational Medicine, Seoul National University College of Medicine, Seoul, Republic of Korea; 4 Interdisciplinary Program in Cancer Biology, Seoul National University College of Medicine, Seoul, Republic of Korea; 5 Department of Biochemistry and Molecular Biology, Seoul National University College of Medicine, Seoul, Republic of Korea; 6 Cancer Research Institute, Seoul National University, Seoul, Republic of Korea

**Keywords:** Cell-free DNA biology, In vitro models, Fragmentomics, Organoids, Extrachromosomal circular DNA

## Abstract

**Background:**

Fragmentomics, the investigation of fragmentation patterns of cell-free DNA (cfDNA), has emerged as a promising strategy for the early detection of multiple cancers in the field of liquid biopsy. However, the clinical application of this approach has been hindered by a limited understanding of cfDNA biology. Furthermore, the prevalence of hematopoietic cell-derived cfDNA in plasma complicates the in vivo investigation of tissue-specific cfDNA other than that of hematopoietic origin. While conventional two-dimensional cell lines have contributed to research on cfDNA biology, their limited representation of in vivo tissue contexts underscores the need for more robust models. In this study, we propose three-dimensional organoids as a novel in vitro model for studying cfDNA biology, focusing on multifaceted fragmentomic analyses.

**Results:**

We established nine patient-derived organoid lines from normal lung airway, normal gastric, and gastric cancer tissues. We then extracted cfDNA from the culture medium of these organoids in both proliferative and apoptotic states. Using whole-genome sequencing data from cfDNA, we analyzed various fragmentomic features, including fragment size, footprints, end motifs, and repeat types at the end. The distribution of cfDNA fragment sizes in organoids, especially in apoptosis samples, was similar to that found in plasma, implying occupancy by mononucleosomes. The footprints determined by sequencing depth exhibited distinct patterns depending on fragment sizes, reflecting occupancy by a variety of DNA-binding proteins. Notably, we discovered that short fragments (< 118 bp) were exclusively enriched in the proliferative state and exhibited distinct fragmentomic profiles, characterized by 3 bp palindromic end motifs and specific repeats.

**Conclusions:**

In conclusion, our results highlight the utility of in vitro organoid models as a valuable tool for studying cfDNA biology and its associated fragmentation patterns. This, in turn, will pave the way for further enhancements in noninvasive cancer detection methodologies based on fragmentomics.

**Supplementary Information:**

The online version contains supplementary material available at 10.1186/s40246-023-00533-0.

## Background

In recent years, cell-free DNA (cfDNA) fragmentomics has emerged as a promising approach for multi-cancer early detection that complements traditional mutation-based liquid biopsy analyses [1, 2, 3, 4]. While mutation-based approaches are limited to the detection of relatively few DNA fragments carrying specific mutations, fragmentomic analysis examines the fragmentation patterns of the entire cfDNA population, providing more comprehensive information, including tissue-of-origin and pathologies associated with altered fragmentomic profiles.

To fully harness its potential for clinical applications, a thorough understanding of the biology underlying cfDNA generation, particularly the fragmentation process of cfDNA, is required [5, 6, 7, 8]. However, investigating cfDNA biology using in vivo samples has been limited due to several challenges. One such challenge is distinguishing between cfDNA fragments derived from tumor cells and those derived from normal cells. Because plasma cfDNA is derived predominantly from hematopoietic cells [9, 10, 11], the “noise” from these cells can obscure the true signals from tumor cells. Another challenging aspect of using in vivo samples is identifying the specific molecular features of cfDNA fragments associated with tumor growth and apoptosis [7, 12]. While apoptosis is considered the main mechanism of cfDNA release [7], it has been suggested that cfDNA can also be released during cell proliferation through dysregulated mitosis [13] or active secretion via extracellular vesicles [14]. However, simulating these biological processes using in vivo models is limited.

In order to overcome these challenges, researchers have used cfDNA isolated from in vitro cell/tissue culture media [15, 16, 17, 18, 19]. In addition to removing the potentially confounding effects of in vivo fragmentation or clearance, this approach enables the manipulation of diverse experimental conditions that can provide a deeper understanding of cfDNA generation at the cellular level. To date, most in vitro studies of cfDNA have been performed using two-dimensional (2D) cell line cultures. However, 2D cell lines do not adequately reflect the in vivo environment because they comprise a single cell type and lack complex cell–cell and cell-extracellular matrix interactions that occur in vivo. In addition, previous studies using 2D cell lines focused primarily on analyzing fragment size by low-resolution capillary electrophoresis. As a result, this approach offered limited insights into the various biological aspects of cfDNA fragmentation.

Recently, three-dimensional (3D) organoids have emerged as promising in vitro models for biomedical research. 3D organoid models mimic the architecture and physiology of the in vivo condition more accurately than 2D cell lines [20]. It has been revealed that cfDNA is present in the media of 3D cultures of preimplantation embryos [21, 22, 23], C3A spheroids [18], and pancreatic cancer organoids [24], making organoid models feasible for in vitro cfDNA research. Nonetheless, recent studies employing 3D organoids have mainly concentrated on mutation detection; consequently, fragmentomic features associated with cfDNA production mechanisms remain poorly understood.

In this study, we introduce 3D organoids as a novel in vitro model to investigate cfDNA biology, focusing on fragmentation patterns. First, we establish patient-derived organoids from normal lung airway tissue, normal gastric tissue, and gastric cancer tissue. We then culture the established organoids in proliferative and apoptotic states simulating the diverse conditions of cfDNA release in vivo. Next, using whole-genome sequencing (WGS) data from cfDNA, we analyze the fragmentomic features of the cfDNA, including fragment sizes, footprinting, end motifs, and repeat types at the end. Finally, using bioinformatic reconstructions of the WGS data, we explore the possibility that a portion of the cfDNA originated from circular DNA.

## Results

### Development of an organoid-based model to investigate tissue-derived cfDNA in vitro

First, we established organoids from both benign and cancerous tissues (Additional file 1: Table S1). In total, nine tissue specimens were collected from nine individuals, three from each of the three types of tissue. We then generated nine organoid lines from each tissue specimen. Specifically, we generated three lung normal organoids (LNO) from non-cancerous lung tissues obtained from lung cancer patients. Additionally, we established three gastric normal organoids (GNO) using gastric tissue collected during sleeve gastrectomy for severely obese patients. Furthermore, we generated three gastric cancer organoids (GCO) using cancerous tissues xenografted onto mice from gastric cancer patients. A detailed process for organoid establishment is provided in the Methods.

**Fig. 1:**
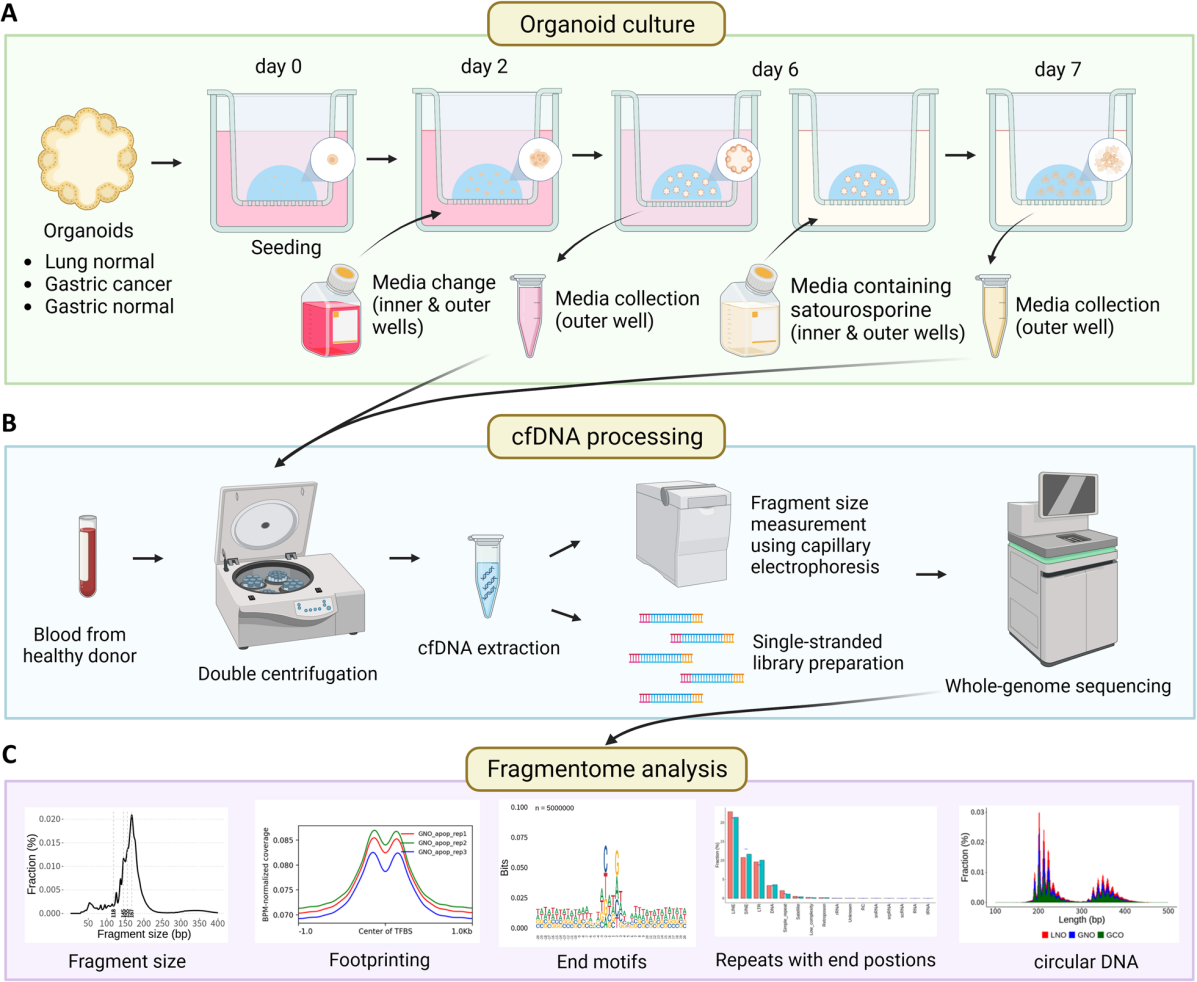
Summary of the methods used to generate 3D organoids and analyze cfDNA samples. **A** Overview of the organoid culture method. Triplicate normal lung, normal gastric, and gastric cancer organoids were seeded onto transwell inserts with a pore size of 0.4 μm, which prevented the transmission of cells. The medium was replaced with Y-27632-free medium after 48 h (day 2). After 96 h of culture in the proliferative state (day 6), the medium was harvested from the outer well and replaced with medium containing 2 µM staurosporine to induce apoptosis. After incubation with staurosporine for 24 h (day 7), the medium was collected from the outer wells. **B** Processing of cfDNA from culture medium and blood. Blood collected from a healthy donor and media from proliferative and apoptotic organoids were subjected to double centrifugation followed by cfDNA extraction. A portion of the cfDNA was used for the measurement of fragment size via capillary electrophoresis, and a portion was used for the preparation of single-stranded libraries. The prepared libraries were subjected to whole-genome sequencing (WGS). **C** WGS data were used to analyze various fragmentomic features, including fragment size, footprints, end motifs, repeat types at the end, and topology (circular DNA)

Contamination of cfDNA preparations with genomic DNA (gDNA) from lysed cells is a common issue [25, 26, 27]. However, in organoid culture, colonies are embedded in a solidified extracellular matrix that prevents their detachment from the plates, minimizing the risk of gDNA contamination. To further reduce the possibility of gDNA contamination, we adopted a transwell culture method in which the pore size of the inserts (0.4 μm) was smaller than the diameter of single cells, thereby preventing cell transfer to the outer well (Fig. 1A). In the subsequent steps, we used only outer well media for cfDNA extraction.

Media from organoid cultures were collected at two time points reflecting tissue proliferation and apoptosis. After 48 h of seeding (day 2), we changed the media to stabilize the organoids and collected media on day 6 that represented the proliferation state. Subsequently, we added medium supplemented with staurosporine to induce apoptosis [28] and obtained media after 24 h of incubation (day 7). Staurosporine-induced apoptosis was confirmed through bleb formation [29] and acridine orange/propidium iodide staining (Additional file 1: Fig. S1). We referred to media obtained on day 6 and day 7 as “proliferation” and “apoptosis”, respectively. A healthy donor blood sample was also collected for comparative analysis (Fig. 1B).

Subsequently, we isolated cfDNA from organoid media and blood using the standard double centrifugation protocol [30]. The Plasma/Serum Cell-Free Circulating DNA Purification Mini Kit (Norgen Biotek) was employed for cfDNA extraction due to its superior performance in extracting short fragments [31]. Capillary electrophoresis revealed nucleosome-sized DNA peaks, with an enrichment of short fragments (< 100 bp) exclusively in proliferation samples (Additional file 1: Fig. S1B–D).

Next, for a total of 19 cfDNA samples extracted from 18 organoid media (proliferation and apoptosis for each of the nine organoid lines) and one plasma sample, single-stranded libraries were prepared using the SRSLY PicoPlus Kit (Claret Bioscience). This kit preserves the native termini of cfDNA fragments, which is important for downstream analysis [32]. We then conducted WGS in a 150 bp paired-end manner with an average coverage of 8x. Using the WGS data, we analyzed fragmentomic features encompassing fragment sizes, footprints, end motifs, repeat types at the end, and topology (Fig. 1C).

### The fragment size of cfDNA from organoids shows enriched short fragments in the proliferation state

First, we investigated the distribution of cfDNA fragment size. We determined fragment sizes using two alternative approaches: read length and insert size. Read length, the number of base pairs sequenced from a DNA fragment, accurately represents the original fragment length but is constrained to 150 bp due to platform (Illumina HiSeq) limitations. Insert size, indicating fragment sizes after alignment to the reference genome, can potentially deviate from the original fragment length due to soft-clipping (exclusion of sequences not aligned to the reference genome) yet offers estimates for fragments longer than 150 bp. Therefore, we used two approaches complementary, employing read length for fragments under 150 bp and insert size for those exceeding this threshold.

**Fig. 2:**
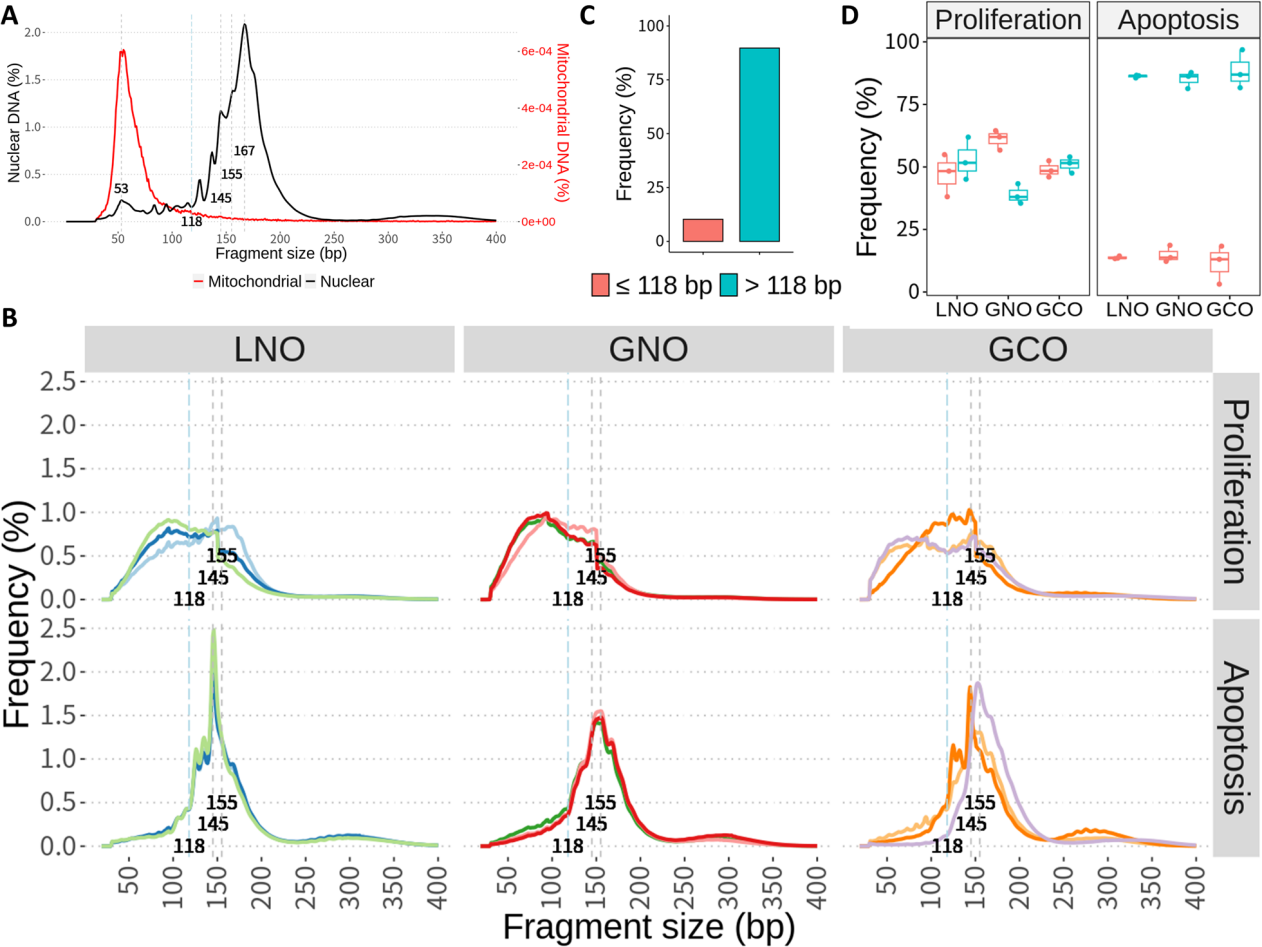
Fragment size analysis of cfDNA. **A** Fragment size distribution of cfDNA in plasma based on the insert size. The black line represents nuclear DNA, and the red line represents mitochondrial DNA. The gray dashed lines and the numbers indicate peak sizes. **B** Fragment size distribution of cfDNA in proliferation and apoptosis organoid samples based on read lengths for fragments less than 150 bp and insert size otherwise. The gray dashed lines and the numbers indicate peak sizes. The light blue long dashed lines and the numbers indicate the cutoff point (118 bp) for separating groups based on fragment size. **C**, **D** The proportions of short (≤ 118 bp) and long (> 118 bp) fragments in plasma (**C**) and organoids (**D**)

The fragment size distribution observed in the plasma was consistent with the well-established profile of healthy plasma [32, 33] (Fig. 2A). Specifically, nuclear DNA exhibited a prominent 167 bp peak with 10 bp periodicity, identical to a previously described mononucleosome occupancy pattern. In addition, as in previous reports that used single-stranded libraries [32], a minor peak was found at 53 bp with no accompanying 10 bp periodicity. In contrast to nuclear DNA, mitochondrial DNA consisted of sub100 bp fragments devoid of oscillations.

In organoids, proliferation and apoptosis samples showed different fragment size distributions of nuclear DNA (Fig. 2B). While apoptosis samples displayed distributions similar to plasma, except for peak sizes at 145 or 155 bp, proliferation samples were enriched for short fragments, which was particularly evident when 118 bp was set as the threshold where the slope of the size distribution changed steeply (Figs. 2C, D). Furthermore, in proliferation samples, a notable decline in size distribution was observed beyond 150 bp, possibly due to the application of different metrics for fragment size beyond this point. To address this, we performed shallow sequencing (0.4 × depth) of three proliferation samples using Illumina MiSeq, with a maximum read length of 300 bp. Based on the read length, the fragment size distribution presented a smooth pattern around 150 bp and remained enriched with short fragments (Additional file 1: Fig. S2).

In conclusion, by analyzing fragment size distribution, we demonstrated that cfDNA from organoid samples in apoptosis had abundant mononucleosomal size similar to plasma samples. On the other hand, cfDNA from organoid samples in proliferation was enriched for short fragments (118 bp), implying distinct biological processes for cfDNA generation.

### DNA-binding proteins have different footprints depending on the cfDNA fragment size

**Fig. 3:**
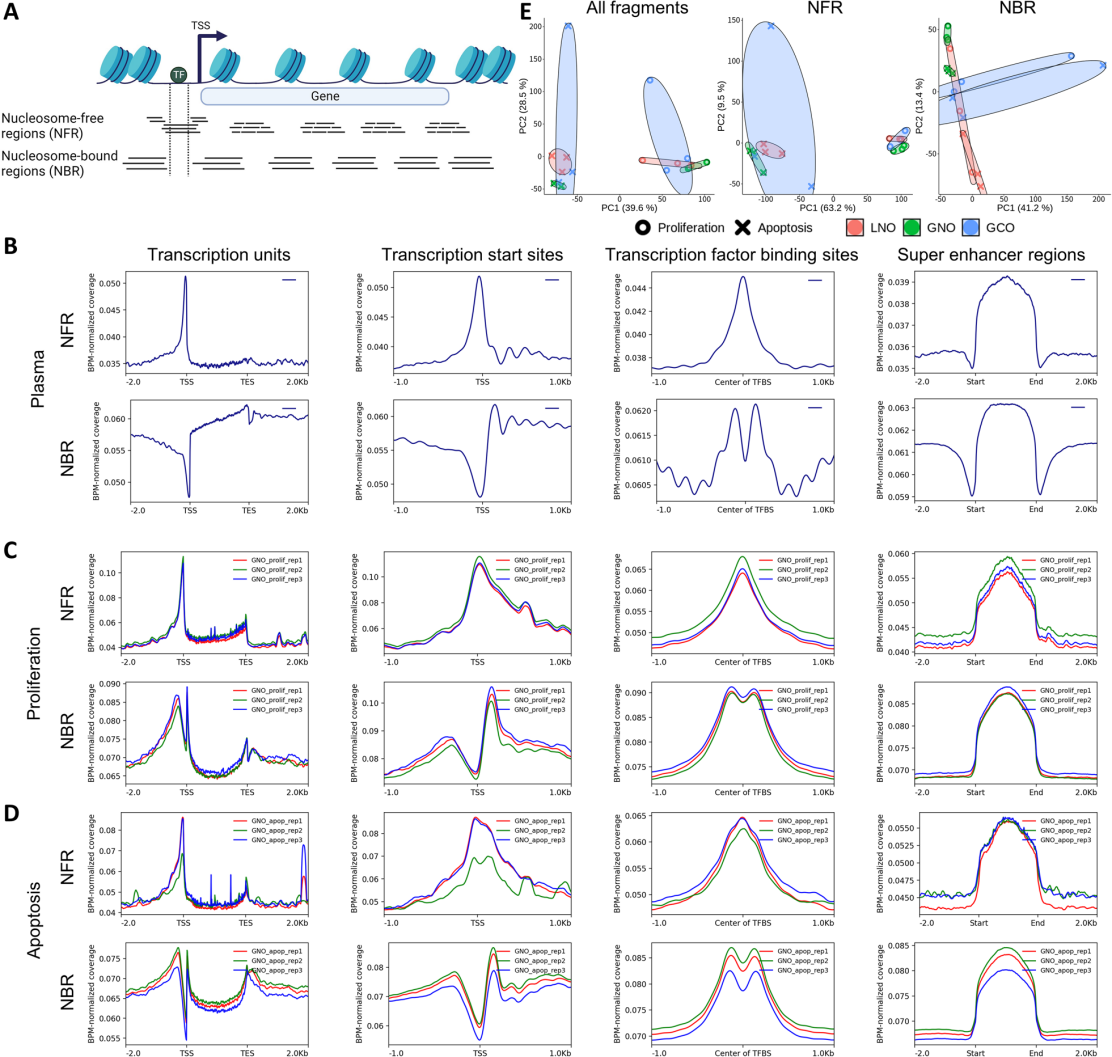
Footprints of DNA-binding proteins in cfDNA. **A** We classified fragments into two groups according to their length, with a cutoff of 118 bp. The shorter fragments were considered to be derived mainly from nucleosome-free regions (NFRs), whereas the longer fragments were considered to be derived mainly from nucleosome-bound regions (NBRs). **B**–**D** Bins per million mapped reads (BPM)-normalized depth around various protein binding regions, including transcription units, transcription start sites (TSSs) or end sites (TESs), transcription factor binding sites (TFBSs), and super enhancer regions, are shown for the plasma sample (**B**) and the proliferation (**C**) and apoptosis (**D**) for GNO samples. **E** Principal component analysis using the normalized depth of transcription units for all fragments, NFR fragments, or NBR fragments. The shape of each datapoint indicates the state (proliferation or apoptosis), and the color indicates the type of organoid (LNO, GNO, or GCO). The ellipses encompass samples of the same type and state

Previous studies have demonstrated that the positioning of cfDNA fragments can represent the footprints of DNA-binding proteins [9, 34, 35, 36]. Specifically, short fragments derived from nucleosome-free regions (NFRs) display footprints of small regulatory proteins such as transcription factors, while long fragments derived from nucleosome-bound regions (NBRs) exhibit nucleosome footprints (Fig. 3A). To explore this phenomenon in our data, we categorized fragments into two groups, “NFR” and “NBR”, setting the cutoff as 118 bp. By analyzing normalized depth enrichments around various protein binding regions—including transcription units (TUs), transcription start sites (TSSs), transcription factor binding sites (TFBSs), and super enhancer regions (SEs)—we observed distinct footprints for both fragment groups (Fig. 3B–D; Additional file 1: Fig. S3).

In the plasma sample, footprints were evident across protein binding regions, with each fragment group showing different characteristics (Fig. 3B). Within TUs, both fragment groups displayed increased enrichment toward transcription end sites, with NBR fragments showing a more prominent pattern. At TSSs and TFBSs, NFR fragments were enriched, whereas NBR fragments were depleted. Additionally, in adjacent regions, NBR fragments displayed periodic nucleosome footprints. In SEs, both fragment groups exhibited similar enrichment patterns, implying densely clustered DNA-binding proteins.

Analysis of organoid samples demonstrated analogous distribution patterns as seen in the plasma sample (Fig. 3C–D; Additional file 1: Fig. S3). However, footprints were less prominent, particularly for NBR fragments. This difference might arise from reduced cfDNA fragmentation in vitro compared to in vivo conditions where circulating endonucleases further cleave cfDNA.

Subsequently, we explored whether the genome-wide enrichment of cfDNA fragments in protein binding regions reflects tissue-specific chromatin accessibility, thus enabling tissue type differentiation. To this end, we generated principal component analysis plots for all fragments, NFR fragments, and NBR fragments using normalized depth in each protein binding region (Fig. 3E; Additional file 1: Fig. S4). Our analysis demonstrated that samples within the same state (proliferation or apoptosis) exhibit distinct clustering, except for NBR fragments in TUs (Fig. 3E). Additionally, in LNO and GNO samples, the same tissue types tended to cluster together. However, GCO samples displayed extensive dispersion, occasionally overlapping with LNO sample distribution, possibly due to highly varying chromatin accessibility among replicates of cancer samples.

Taken together, our findings suggest that cfDNA from our organoid model may reflect differences in chromatin accessibility according to cell states (proliferation or apoptosis) and normal tissue types.

### NFR fragments from proliferation samples show distinct end motifs

The specific end motifs of cfDNA fragments are influenced by the endonuclease responsible for their cleavage during generation [5, 6, 37, 38]. Our investigation revealed that short cfDNA fragments were highly abundant only in proliferation samples. This finding suggests that different biological processes may be involved in the generation of short fragments compared to long fragments. To explore this hypothesis, we analyzed the end motifs of NFR and NBR fragments leveraging the advantage of ssDNA library preparation that enables preservation of native termini.

**Fig. 4:**
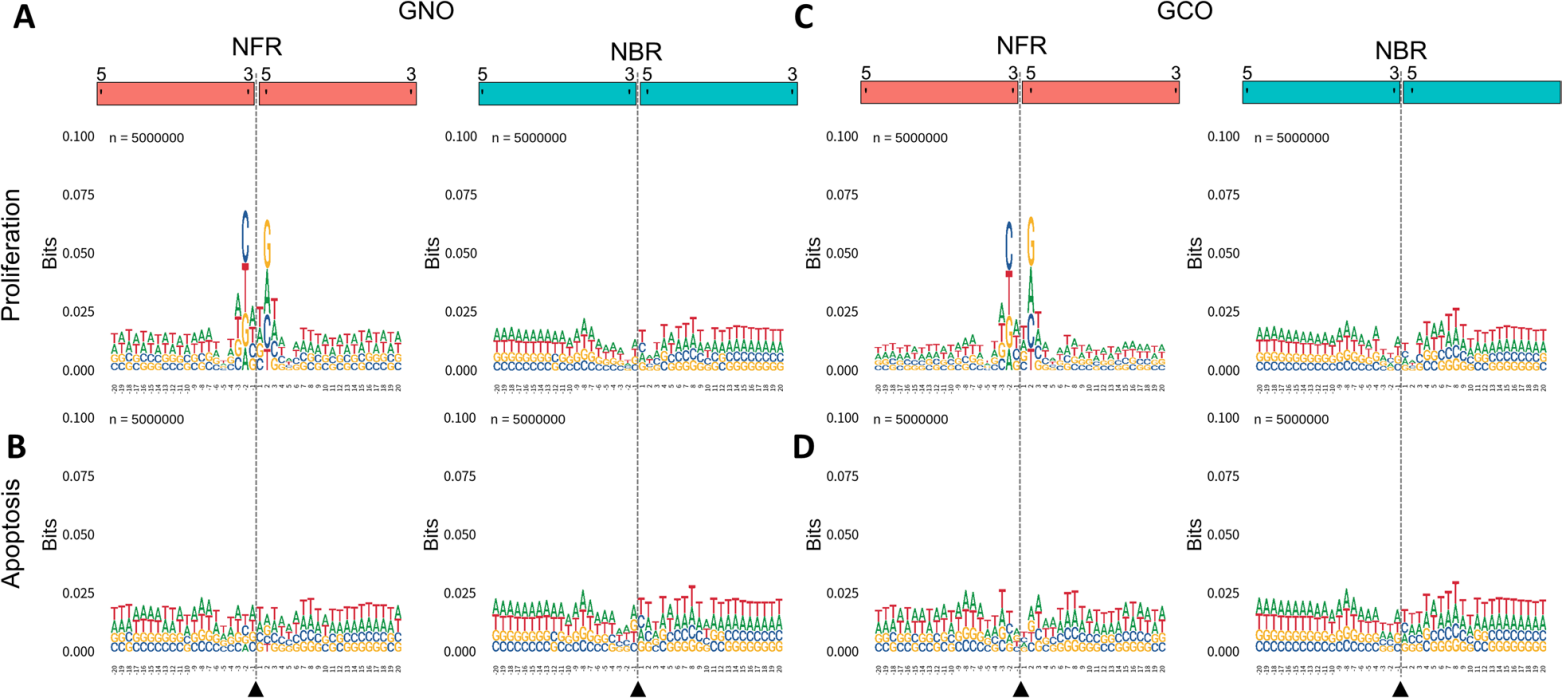
End motifs of cfDNA fragments. End motifs were constructed using 5 million randomly selected NFR and NBR fragments. Breakpoints were defined as the points between the 3′ and 5′ ends and are indicated by gray dashed lines and triangle symbols. Representative results for GNO (**A** and **B**) and GCO (**C** and **D**) samples in the proliferative (**A** and **C**) and apoptotic (**B** and **D**) states are shown. Data were similar for other tissue types (Additional file 1: Fig. S5)

First, we randomly selected 5 million fragments per sample from the FASTA files to normalize sequencing depth differences. We then analyzed nucleotide sequences adjacent to the 5′ and 3′ termini to reveal DNA motifs (Fig. 4; Additional file 1: Fig. S5). As a result, NFR fragments from proliferating organoids displayed a significant enrichment of a palindromic 3 bp motif near the fragment ends. In contrast, no such enrichment was observed for NBR fragments. Furthermore, this characteristic palindromic motif was absent in both NFR and NBR fragments from apoptotic organoids, as well as in the plasma sample. These findings suggest that distinct biological mechanisms may contribute to the formation of NFR fragments, especially during cellular proliferation.

### NFR fragments from proliferation samples have distinct repeat patterns at the fragment ends

**Fig. 5:**
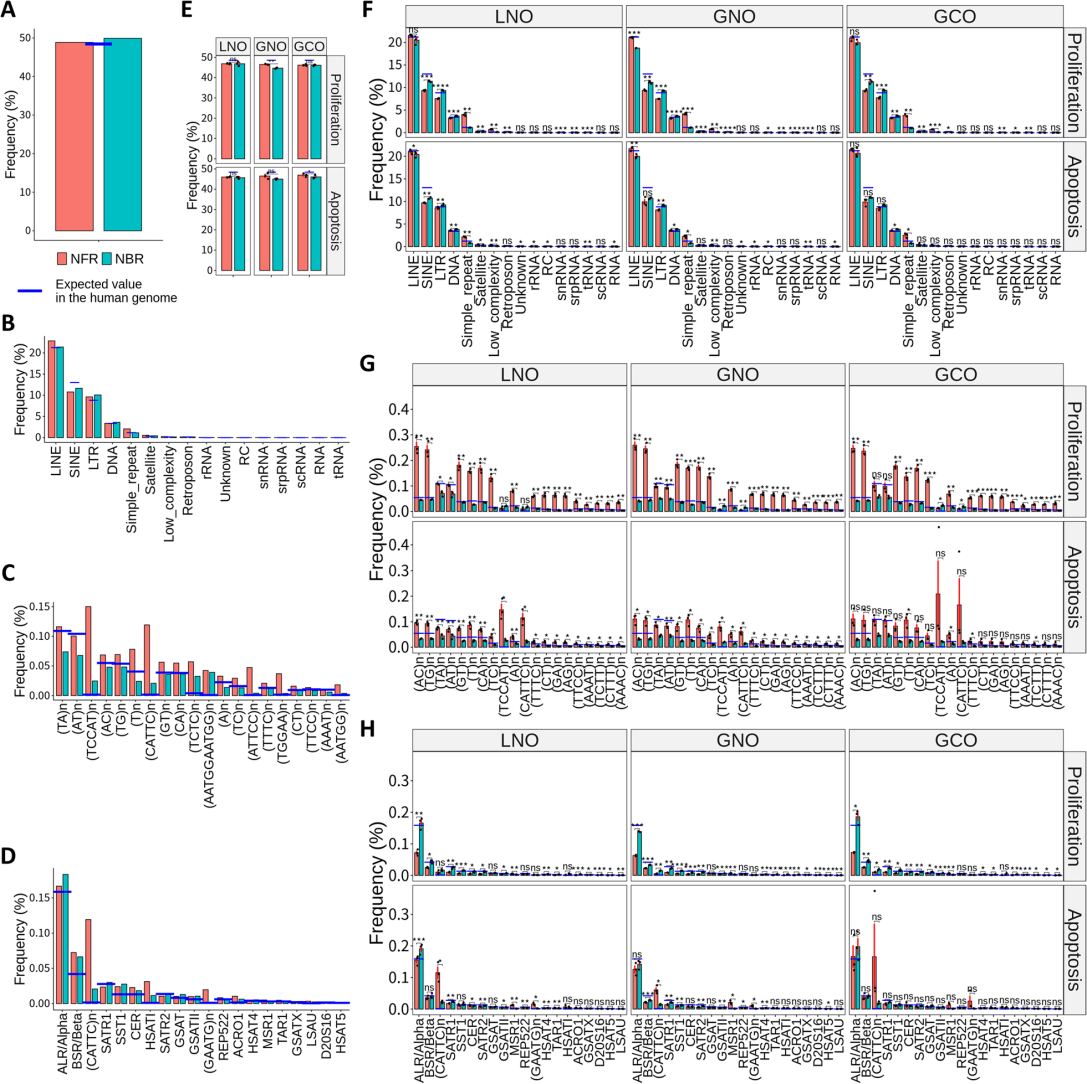
Proportions of the end positions of NFR and NBR fragments in repeat regions. **A** The proportion of fragment ends from the plasma sample in entire RepeatMasker-defined repeat regions. **B**–**D** The proportion of fragment ends from the plasma sample in RepeatMasker-defined repeat classes (**B**), subclasses of simple repeats (**C**), and subclasses of satellite repeats (**D**). **E** The proportion of fragment ends from organoid samples in entire RepeatMasker-defined repeat regions. **F**–**H** The proportion of fragment ends from organoid samples in RepeatMasker-defined repeat classes (**F**), subclasses of simple repeats (**G**), and subclasses of satellite repeats (**H**). Blue horizontal bars represent the proportions of RepeatMasker-defined repeats in the human genome. Black dots represent the value for each replicate. Red error bars represent the standard error of the mean. Statistical analyses were performed using the R package rstatix with Welch’s *t* test (ns: not significant; *p < 0.05, **p < 0.01, ***p < 0.001, and ****p < 0.0001)

A previous study revealed that cfDNA fragments containing specific microsatellite repeats (simple repeats) were shorter than genome-wide fragments [33]. Building upon this study and our discovery that NFR and NBR fragments possess distinct end motifs, we hypothesized that their end positions might lie within different repeat regions. To explore this hypothesis, we calculated the proportion of end positions in RepeatMasker-defined repeats for both fragment groups, as well as expected values in the human genome (Fig. 5).

In the plasma sample, the proportion of fragment ends in entire repeat regions was similar to the expected proportion in the human genome, with a slightly higher proportion in NBR fragments (Fig. 5A). The proportion of each repeat class was also similar to the expected values, with slight differences between fragment groups (Fig. 5B). For each repeat class, we then analyzed the proportion of repeat subclasses. There was a slight difference between fragments of NFR and NBR in LINE and SINE (Additional file 1: Fig. S6A–B). In contrast, when analyzing simple repeats and satellite repeats, there were remarkably higher frequencies of specific repeats in NFR fragments, such as (TCCAT)n and (CATTC)n (Fig. 5C–D). Moreover, NFR fragments exhibited higher frequencies of low-complexity repeats (Additional file 1: Fig. S6C). However, a statistical comparison was not feasible due to the limited sample size.

For organoid samples, the proportion of fragment ends in entire repeat regions was lower than anticipated in both fragment groups (Fig. 5E). Generally, both the apoptosis and proliferation samples displayed similar distributions of repeat classes to the plasma sample (Fig. 5F). We further analyzed the subclasses for each repeat class (Fig. 5G–H; Additional file 1: Fig. S6D–F). While apoptosis samples exhibited similar patterns to the plasma sample, proliferation samples showed a prominent prevalence of dinucleotide repeats for NFR fragments. Interestingly, repeats such as (TCCAT)n and (CATTC)n, which were abundant in NFR fragments from apoptosis samples, were scarce in proliferation samples.

In summary, our study revealed different distributions of repeat types at fragment ends between proliferation and apoptosis samples. These findings, coupled with our prior discovery of characteristic end motifs in proliferation samples, indicate distinct biological mechanisms contributing to cfDNA fragment generation in different cellular states.

### Circular DNA is a source of cfDNA

Recently, it has been reported that extrachromosomal circular DNA (eccDNA) is found in various healthy tissues, cancers, and even plasma [39, 40, 41, 42, 43]. Thus, we hypothesized that cfDNA could originate from eccDNA alongside linear DNA. To test this, we employed a specialized bioinformatics pipeline using ATAC-seq or WGS data for circular DNA detection [44]. This pipeline leverages split reads from Tn5 tagmentation (ATAC-seq) or sonication (WGS) to reconstruct circular DNA. Since our WGS data have the same information generated by cfDNA fragmentation, we could apply this pipeline.

**Fig. 6:**
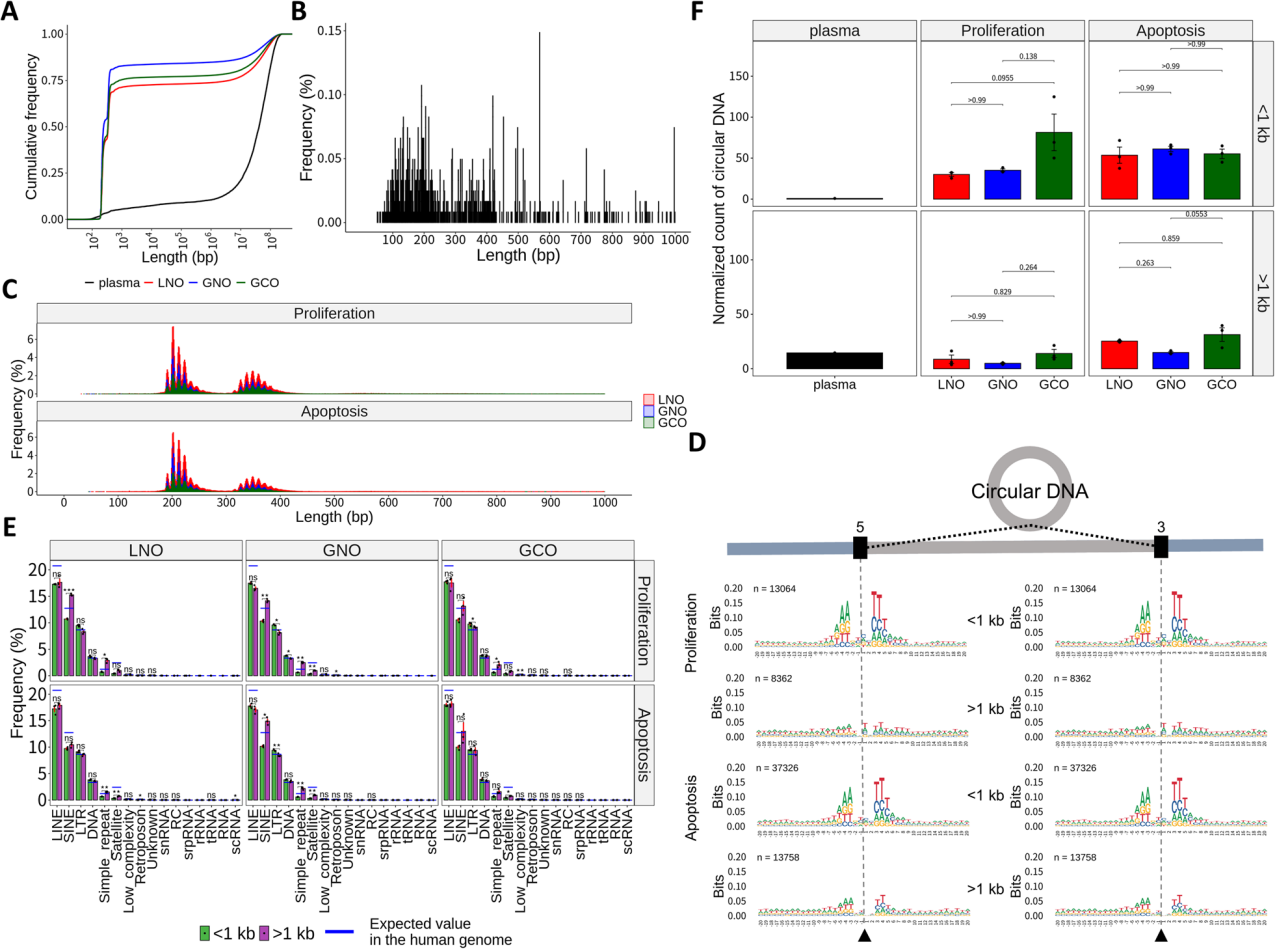
Reconstruction of circular DNA from cfDNA WGS data. **A** Cumulative proportions of circular DNA lengths in the plasma and organoid samples. **B**, **C** Length distribution analysis of circular DNA shorter than 1 kb in the plasma sample (**B**) and the organoid samples (**C**). **D** Circular DNA motifs around junction breaks according to cell states and circular DNA sizes in an LNO sample. Similar results were obtained for other replicates (data not shown). Junction breaks are indicated by gray dashed lines and triangle symbols. **E** The proportion of junction breaks in RepeatMasker-defined repeat classes for the organoid samples. Blue horizontal bars represent the proportions of RepeatMasker-defined repeats in the human genome. Red error bars represent the standard error of the mean. **F** Comparison of the number of circular DNA (counts per million mapped reads) for each sample type. Statistical analyses were performed using the R package rstatix, with Welch’s *t* test or a pairwise *t* test with Bonferroni correction (ns: not significant; *p* < 0.05 and *p < 0.01)

As a result, numerous circular DNA were detected, particularly enriched in short (under 1 kb) or long (over 1 Mb) lengths (Fig. 6A). The plasma sample showed a prevalence of long circular DNA, whereas the organoid samples exhibited a prevalence of short circular DNA. Short circular DNA under 1 kb are typically referred to as microDNA and are considered to have functional properties that are distinct from those of longer circular DNA [40, 45, 46]. High-resolution analysis of short lengths showed characteristic microDNA size distributions [39, 47], with distinct peaks at approximately 202 bp and 349 bp and a 10 bp oscillation, which were obviously evident in organoid samples (Fig. 6B–C).

To explore the characteristics of circular DNA depending on their size, we categorized fragments based on a 1 kb cutoff. Next, we analyzed DNA motifs around junctions within circular DNA, which correspond to terminal sequences from originating linear DNA. In both proliferation and apoptosis samples, we observed dual-repeat patterns in 5′ and 3′ junctions, which were prevalent only in circular DNA under 1 kb (Fig. 6D; Additional file 1: S7A–B). Interestingly, this finding aligns with known junctional motifs of microDNA present in plasma [39].

Next, we investigated the proportion of RepeatMasker-defined repeat regions around junctions. As a result, we observed distinct patterns according to circular DNA length (Fig. 6E; Additional file 1: Fig. S8). In the plasma, circular DNA over 1 kb was enriched in LINEs and SINEs, while circular DNA under 1 kb exhibited higher enrichment in simple repeats and satellites (Additional file 1: Fig. S8B). In organoids, proliferation and apoptotic samples showed similar patterns and were different from the plasma, such as no difference in the proportion of LINEs according to circular DNA size (Fig. 6E).

Previous studies have shown that cancer cells have more circular DNA than normal cells [41, 42, 47]. To evaluate this in our data, we calculated the circular DNA count in each sample using the number of sequencing reads contributing to circular DNA reconstruction, normalized to per million mappable reads (Fig. 6F). Although statistical significance was not achieved, likely due to high variability among GCO replicates, GCO samples had a higher content of circular DNA, except for circular DNA under 1 kb in the apoptosis samples.

Since we successfully reconstructed circular DNA with the known profile of eccDNA from cfDNA fragments, we can infer that parts of cfDNA are derived from circular DNA. Our results, although limited to bioinformatic analysis, suggest potential associations between cfDNA and eccDNA.

## Discussion

In this study, we presented a novel method for examining cfDNA biology in vitro using organoids, focusing on fragmentomic analysis. Employing organoids enabled us to examine cfDNA derived from diverse tissues in different states of proliferation and apoptosis. Furthermore, this approach allowed us to compare normal and cancerous tissues, which is not possible with conventional 2D cell lines that consist only of immortalized cells. Although we could not observe significant differences between normal and cancer organoids, we revealed that short fragments (< 118 bp) with distinct fragmentomic features were generated exclusively during proliferation. This novel discovery underscores the usefulness of organoids as a promising in vitro model for cfDNA research.

Previous studies have demonstrated that the size of cfDNA is associated with DNA-binding proteins that protect against cleavage [9, 48, 49, 50, 51, 52, 53, 54]. While hematopoietic cell-derived cfDNA typically exhibits a peak size of 167 bp, cfDNA from various sources, such as fetuses, donor livers, and certain types of cancer, displays relatively short peak sizes ranging from 135 to 155 bp [48, 49, 50, 51, 52, 53, 54]. Despite the presumed association with tissue-specific chromatin accessibility, the precise biological factors contributing to these fragment size differences across tissue types remain unclear. In this study, we observed that fragment sizes were shorter in organoids than in plasma, with peak sizes ranging from 145 to 155 bp. While the detailed mechanism governing this difference in cfDNA size was not explored in depth in our study, further investigations using our organoid model can provide insight into this mechanism.

Recently, it was demonstrated that single-stranded cfDNA fragments as short as approximately 50 nt can be detected in plasma by optimizing cfDNA extraction and ssDNA library preparation [55, 56, 57]. We also found that cfDNA fragments shorter than 118 bases were enriched during proliferation in organoid samples. However, we believe that the short fragments identified in our study were composed primarily of double-stranded DNA for two reasons: first, the cfDNA extraction kit we used cannot extract single-stranded DNA efficiently [55]; and second, capillary electrophoresis, which only detects double-stranded DNA, revealed enrichment of these short fragments. To our knowledge, this class of cfDNA has never been described before and can only be discovered through our in vitro models that simulate proliferation.

In healthy plasma, although we detected a small peak at 53 bp, we could not detect a similar enrichment pattern of short fragments as in proliferating organoids. It is possible that although short fragments from proliferating hematopoietic stem cells in the bone marrow do exist, they might undergo further fragmentation or clearance within the bone marrow microenvironment or circulation. Additionally, a high excess of nucleosome-sized fragments from apoptotic cells in the peripheral blood could prevent the detection of short fragments. Meanwhile, in cancer patients, there may be an excessive release of short fragments from rapidly dividing malignant cells, allowing them to be detected. This assumption is supported by clinical studies on pancreatic cancer patients that specifically detected cfDNA derived from cancer cells. These studies utilized mutation-specific targeted amplicons [58] or capture sequencing [59] to effectively enrich a small amount of cancer-derived cfDNA. In these studies, cancer-derived cfDNA containing KRAS hotspot mutations were enriched in ultrashort sizes (< 100 bp). Notably, in the study by Liu et al., the median lengths of mutated fragments in patients with precancerous (intraductal papillary mucinous neoplasm), early, and advanced stage pancreatic cancer were approximately 80, 140, and 160 bp, respectively, indicating that shorter fragments were released at earlier stages [59]. In contrast, they observed that the wild-type fragments assumed to originate from hematopoietic cells had a length of approximately 160 bp across all subgroups. To explain this unprecedented finding, they hypothesized that in early-stage cancers, short cfDNA fragments are generated due to widespread DNA damage by apoptosis and immune clearance mechanisms, effectively captured by single-stranded library preparation. On the other hand, in late-stage cancers, they postulated that large fragments might result from hypoxia-related necrosis. However, it is still unclear how cfDNA is generated and released by cancer cells throughout the course of their growth, as well as how cfDNA is cleared. Therefore, we believe that our finding that proliferating cells predominantly release short fragments (< 118 bp) has substantial biological implications, possibly explaining previous reports of ultrashort cfDNA in early-stage cancers.

In recent years, there has been growing interest in exploring the biogenesis of eccDNA [46, 60, 61]. Based on a previously validated bioinformatic approach [44], we detected eccDNA using cfDNA WGS data. Our in vitro model did not involve exogenous nucleases; therefore, we hypothesized that a portion of the extracted cfDNA might have been generated via the fragmentation of circular DNA by cellular endonucleases. Interestingly, our analysis revealed that short circular DNA (< 1 kb) from organoid samples had distinct motifs around junction breaks, which were identical to known motifs of microDNA. Although further research is necessary to establish functional evidence for these results, our study provides additional insight into the biology of eccDNA generation in relation to cfDNA.

## Conclusions

In summary, we propose 3D organoids as a novel in vitro model system for cfDNA research. Using this system, we demonstrated that short (< 118 bp) fragments released from proliferating tissues possess distinct fragmentomic features, offering valuable insights into cfDNA generation. As a proof-of-concept, we studied only a small number of tissue types and conditions focusing on fragmentomics. Further experiments involving more tissue types and varying manipulations will, however, provide a better understanding of cfDNA biology. Finally, we expect that the practical implications of our results in the context of implementing multi-cancer early detection strategies will be validated in future studies.

## Methods

### Blood sample processing

We collected blood from a healthy donor using an EDTA tube. Pure plasma without DNA contamination from blood cells was isolated using a two-step centrifugation process, as recommended by the standard cell-free DNA preparation protocol [30]. The initial centrifugation was performed at 1600 g and 4 °C for 10 min to remove cells present in the buffy coat. The second centrifugation step was performed at 16,000 g and 4 °C for 10 min to eliminate cell organelles and debris. The supernatant plasma was stored at 4 °C until the extraction of cfDNA.

### Organoid establishment

We developed organoids using adult stem cells derived from lung airway, normal gastric, and gastric cancer tissues (Additional file 1: Table S1). To generate LNOs, we collected distal lung tissues from patients undergoing lung resection surgery and used the lung tissues that were farthest from the tumor area. To generate GNOs, we collected gastric tissue from severely obese patients who underwent sleeve gastrectomy at Seoul Slime Surgery. To generate GCOs, we used tissue from patient-derived xenografts established by the research team of Cho et al. [62]. Finally, we established and passaged LNOs, GNOs, and GCOs in triplicate using conventional adult stem cell-derived organoid culture methods (refer to Additional file 1: Supplemental Methods for further details).

### Organoid culture for cfDNA collection

Organoids were dissociated into single cells by manual pipetting once before, once during, and once after incubation in TrypLE Express for 10 min at 37 °C. Cells were counted using Trypan blue dye and a Countess II Automated Cell Counter (Thermo Fisher Scientific) at the Genomic Medicine Institute Research Service Center, and then 50,000 cells were resuspended in 40 µL of Cultrex™ Basement Membrane Extract (R&D Systems). These resuspended cells were seeded onto inserts of a 12-well Transwell plate with a 0.4 µm pore size (Corning), using eight wells per tissue type. After incubating at 37 °C for 20 min, 0.9 mL and 0.4 mL of Y-27632-supplemented medium that was optimized for each tissue type was added to the outer and inner wells, respectively. On day 2, 48 h after seeding the organoids, the medium was changed to Y-27632-free medium. On day 6, the medium was harvested from the outer wells, and the medium from the inner wells was discarded. To eliminate cfDNA generated during organoid proliferation, the wells were washed three times with PBS, and then medium containing 2 µM staurosporine was added to the inner wells (0.4 mL) and outer wells (0.9 mL). The medium was harvested 24 h later (day 7). The medium from day 6 and day 7 was centrifuged twice and then stored at 4 °C until cfDNA was extracted.

### Extraction of cfDNA and fragment size measurement using capillary electrophoresis

The Plasma/Serum Cell-Free Circulating DNA Purification Mini Kit (Norgen Biotek) was used to extract cfDNA from media collected on days 6 and 7. The concentration of the extracted double-stranded DNA was determined using a Qubit Fluorometer (Invitrogen). To measure the fragment size of each sample, we used an Agilent 2100 Bioanalyzer (Agilent Technologies) at the Genomic Medicine Institute Research Service Center, along with the Agilent High Sensitivity DNA Kit, which is based on capillary electrophoresis.

### Library preparation and WGS

To prepare sequencing libraries from cfDNA, we first removed fragments larger than 1000 bp using SPRIselect beads (Beckman Coulter) because the Illumina short-read sequencing platform cannot recover these fragments. The SRSLY PicoPlus Kit (Claret Bioscience) was used to prepare single-stranded DNA libraries according to the manufacturer’s instructions, enabling us to recover both double-stranded DNA molecules with nicks and single-stranded DNA molecules [32]. The libraries were then whole-genome sequenced in 150 bp paired-end mode with a depth of coverage of 18× for the plasma sample and 3–13× for the organoid samples using the NovaSeq6000 system (Macrogen Inc.). For each organoid type, a single sample was further sequenced in 300 bp paired-end mode with a depth of coverage of approximately 0.4× using MiSeq. We processed the raw FASTQ files with fastp (https://github.com/OpenGene/fastp) [63] by performing adapter trimming based on per-read overlap analysis, which identifies the overlap of each pair of reads and preserves the original sequences for fragments shorter than 150 bp. The trimmed FASTQ files were aligned to the hg38 reference genome using the BWA-MEM algorithm, and duplicate reads were removed using Picard MarkDuplicate. Finally, we excluded reads that overlapped with the ENCODE Blacklist (version 3) [64] using SAMtools. All bioinformatics analyses were performed using the computing server at the Genomic Medicine Institute Research Service Center.

### Fragment size analysis

Picard CollectInsertSize was used to determine the distribution of insert sizes. However, due to soft-clipping of mismatched sequences, the actual fragment length can be underestimated, particularly for short fragments (< 150 bp). To overcome this issue, we used the original read length as the fragment size for reads shorter than 150 bp. The “awk” command in bash was used to extract the read length from the BAM file. As a result, we noticed that the fragment size distribution changed markedly at approximately 118 bp. Therefore, we divided the fragments into two groups: NFR (fragments smaller than or equal to 118 bp) and NBR (fragments larger than 118 bp). For each sample, we generated two BAM files corresponding to NBR and NFR using SAMtools.

### Footprinting of DNA-binding proteins

First, the BAM files for NFR and NBR were converted into bigwig files with 10 bp bins using the bamCoverage command of the deepTools package [65]. We normalized the sequencing depth as bins per million mapped reads (BPM), which is equivalent to transcripts per million transcripts (TPM) in RNA-seq. During this process, reads with a mapping quality score lower than 30 were excluded. Next, we used deepTools computeMatrix to calculate the normalized sequence depths around known protein binding sites, including TUs, TSSs, TFBSs, and SEs. The database of TUs and TSSs was obtained from the RefSeq GTF file, and TFBSs were obtained from ENCODE Regulation TF Clusters, which were downloaded from the UCSC genome annotation database. In addition, we used a database containing approximately 377 Mb SEs from 86 human cell and tissue types [66]. Finally, we generated a plot of the enrichment around these sites using deepTools plotProfile based on the resulting matrix.

### End motif analysis

DNA sequences were extracted in FASTA file format from the NFR and NBR BAM files. For paired reads, we obtained sequences near the 5' end of the first read and the 3' end of the second read. To ensure that the analysis was not biased by varying sequencing depths across samples, we randomly selected 5,000,000 sequence pairs from each sample. Finally, we used the R ggseqlogo package (https://github.com/omarwagih/ggseqlogo) to generate DNA sequence motifs from the extracted sequences.

### Analysis of end positions in repeats

First, we converted the NFR and NBR BAM files into BED files, which represent the mapped regions on the reference genome, using the bedtools bamtobed tool. From these BED files, we extracted the end positions of reads near the 5' end. Using bedtools intersect, we annotated the repeat family/class for these end positions using the RepeatMasker annotation downloaded from the UCSC genome annotation database. Finally, we analyzed the distribution of repeat regions using our in-house Python and R scripts.

### Circular DNA analysis

Circle_finder (https://github.com/pk7zuva/Circle_finder), a tool originally developed for ATAC-seq or WGS libraries [44], was used to identify circular DNA in the WGS data. This pipeline can detect circular DNA without requiring its physical enrichment by analyzing junction sequences created by DNA ligation into circular forms that are not present in the normal reference genome. We applied this pipeline to our WGS data as the cfDNA is fragmented by endonucleases, similar to Tn5 tagmentation or sonication. To minimize false positive results, known tandem duplication regions were excluded from the analysis since Circle_finder cannot distinguish between tandem duplication and junction sequences. Tandem duplication regions were obtained from the Database of Genomic Variants downloaded from the UCSC genome annotation database.

### Statistical analysis

Comparison of the proportions of repeats between two fragment length groups was performed using a two-sided Welch’s *t* test. A pairwise *t* test with Bonferroni correction was used to compare the counts of circular DNA across organoid types. All statistical analyses were performed using the R package rstatix (https://github.com/kassambara/rstatix).

### Supplementary Information

**Additional file 1. Supplemental Methods** for gastric cancer organoid establishment primary organoid culture and passaging. **Table S1.** Summary of the 3D organoid samples used in the study. **Fig. S1.** Comparison of the proliferation and apoptosis 3D organoid samples. **Fig. S2.** Distributions of the cfDNA fragment sizes sequenced using MiSeq. **Fig. S3.** Footprints of DNA-binding proteins in cfDNA from organoid samples. **Fig. S4.** Principal component analysis using bins per million mapped reads (BPM)-normalized depths for regions of DNA-binding proteins. **Fig. S5.** End motifs of cfDNA fragments. **Fig. S6.** Proportions of the end positions of NFR and NBR fragments in repeat regions. **Fig. S7.** Analysis of DNA motifs surrounding circular DNA junctions. **Fig. S8.** Distribution of junction breaks of circular DNA in repeat regions.

## Data Availability

The dataset supporting the conclusions of this article is available in the NCBI BioProject database under accession number PRJNA954988 (https://www.ncbi.nlm.nih.gov/bioproject/PRJNA954988).

## Abbreviations

BAM: Binary alignment map
cfDNA: Cell-free DNA
eccDNA: Extrachromosomal circular DNA
gDNA: Genomic DNA
GNO: Gastric normal organoids
GCO: Gastric normal organoids
LINEs: Long interspersed nuclear elements
LNO: Lung normal organoids
NBRs: Nucleosome-bound regions
NFRs: Nucleosome-free regions
SAM: Sequence alignment map
SEs: Super enhancer regions
SINEs: Short interspersed nuclear elements
TFBSs: Transcription factor-binding sites
TPM: Transcripts per million transcripts
TSSs: Transcription start sites
TUs: Transcription units
WGS: Whole-genome sequencing

## Code availability

The code used in this study is available at GitHub (https://github.com/jaeryuk/cfDNA-organoid).
