# Supplementary material for: Multidimensional fragmentomic profiling of cell-free DNA released from patient-derived organoids
Source: Hum Genomics. 2023 Oct 28;17:96. doi: 10.1186/s40246-023-00533-0 (PMC10613368; doi:10.1186/s40246-023-00533-0)
Supplement: Supplementary file 1 — Additional file 1. Supplemental Methods for gastric cancer organoid establishment primary organoid culture and passaging. Table S1. Summary of the 3D organoid samples used in the study. Fig. S1. Comparison of the proliferation and apoptosis 3D organoid samples. Fig. S2. Distributions of the cfDNA fragment sizes sequenced using MiSeq. Fig. S3. Footprints of DNA-binding proteins in cfDNA from organoid samples. Fig. S4. Principal component analysis using bins per million mapped reads (BPM)-normalized depths for regions of DNA-binding proteins. Fig. S5. End motifs of cfDNA fragments. Fig. S6. Proportions of the end positions of NFR and NBR fragments in repeat regions. Fig. S7. Analysis of DNA motifs surrounding circular DNA junctions. Fig. S8. Distribution of junction breaks of circular DNA in repeat regions. [file 40246_2023_533_MOESM1_ESM.docx]

**Supplemental Methods**

**Gastric cancer organoid establishment**

To generate GCOs, we used tissue from patient-derived xenografts established by the research team of Cho et al [79]. The stored xenograft tissues were re-transplanted subcutaneously into the flanks of 6-week-old nonobese diabetic/severe combined immunodeficiency/interleukin 2γ-receptor null female mice (The Jackson Laboratory). Once the tumor volumes reached 700–1000 mm^3^, we euthanized the mice and resected the tumor tissues. All fresh tissues were maintained in ADF+++ medium (Advanced DMEM/F12 (Gibco), 1% HEPES (Gibco), 1% Glutamax (Gibco)) containing 0.125 µg/mL amphotericin B (Gibco) and were stored at 4°C for less than 12 hr before processing.

**Primary organoid culture and passaging**

To establish primary LNOs, lung tissues were cut into pieces measuring less than 5 mm and were washed with ice-cold PBS to remove mucus and blood cells. Next, the tissue pieces were dissociated into single cells using a gentleMACS Octo Dissociator with Heater (Miltenyi Biotec), following the manufacturer’s protocol. The resulting tissue suspensions were then filtered through a 70 μm cell strainer (Miltenyi Biotec) and washed with 5 mL of ADF+++. The cells were then centrifuged at 400 g and 4°C for 10 min, and the supernatant was removed. Subsequently, the cell pellets were resuspended in 1 mL of red blood cell lysis buffer (Miltenyi Biotec) and incubated at room temperature for 10 min. The reaction was quenched by adding 9 mL of ADF+++, followed by centrifugation at 400 g and 4°C for 10 min. The resulting cell pellets were resuspended in Growth Factor Reduced Matrigel (GFR-Matrigel; Corning) and plated as 40 μL droplets in a pre-warmed 24-well tissue culture plate. The plates were incubated at 37°C for 15 min and then submerged into 500 μL of pre-warmed airway organoid medium supplemented with 10 µM Y-27632 for the first 3 days. The medium was changed every 3 days. Established 3D airway organoids were passaged at a 1:4 ratio every 1–2 weeks. For passaging, TrypLE Express (Gibco) was added to each well and the GFR-Matrigel was disrupted mechanically and incubated at 37°C for 5 min. The reaction was quenched by adding ice-cold ADF+++, and the cells were centrifuged at 400 g and 4°C for 5 min. Finally, the cells were resuspended in GFR-Matrigel and plated as described above.

To establish primary GNOs, normal gastric tissues were cut into 1 cm^2^ pieces. The mucus layer was carefully separated from the muscle layer and the resulting tissue pieces were cut into pieces smaller than 5 mm and washed with ice-cold DPBS to remove any residual debris. Next, the tissue pieces were transferred to a 10 cm dish containing 10 mL of Gentle Cell Dissociation Reagent (STEMCELL Technologies) and were incubated at room temperature on a rocker at 20 rpm for 20 min. Subsequently, the tissue pieces were allowed to settle by gravity and the supernatant was gently pipetted off. The tissue pieces were then resuspended in 5 mL of ice-cold DPBS and transferred to another 10 cm dish. Using a cover slip, gentle pressure was applied to the tissue pieces until crypts were released and visible in the supernatant. The supernatant containing the crypts was collected and centrifuged at 400 g and 4°C for 5 min. The resulting cell pellets were resuspended in 1 mL of red blood lysis buffer and incubated at room temperature for 10 min. The reaction was quenched by adding 9 mL of ADF+++ and the cells were centrifuged again at 400 g and 4°C for 10 min. Finally, the organoids were seeded onto plates as described for lung organoids, with the exception that gastric organoid medium was used.

To establish primary GCOs, tissues were collected from mouse xenografts and were enzymatically dissociated by incubating in 10 mL of ADF+++ containing 1.5 mg/mL collagenase (Gibco) and 20 mg/mL hyaluronidase (Sigma) for 1 hr at 37°C with shaking. After enzymatic dissociation, the primary seeding and passaging steps were similar to those described for the lung organoids, with the exception that gastric organoid medium was used. Once the primary culture was stabilized (after 1–2 passages), the mouse cells were removed using the Mouse Cell Depletion Kit (Miltenyi Biotec), followed by further passaging.

**Table S1. Summary of the 3D organoid samples used in the study.**

| Library number | Sample name | Patient sex | Patient age (years) | Cell state | Tissue of origin | WGS depth (×) |
| --- | --- | --- | --- | --- | --- | --- |
| 1 | LNO_prolif_rep1 | Male | 52 | Proliferation | Lung airway epithelium | 7.7 |
| 2 | LNO_apop_rep1 |  |  | Apoptosis |  | 12.7 |
| 3 | LNO_prolif_rep2 | Female | 64 | Proliferation |  | 5.5 |
| 4 | LNO_apop_rep2 |  |  | Apoptosis |  | 12.9 |
| 5 | LNO_prolif_rep3 | Female | 56 | Proliferation |  | 4 |
| 6 | LNO_apop_rep3 |  |  | Apoptosis |  | 8.6 |
| 7 | GNO_prolif_rep1 | Female | 28 | Proliferation | Gastric epithelium | 3.1 |
| 8 | GNO_apop_rep1 |  |  | Apoptosis |  | 8.5 |
| 9 | GNO_prolif_rep2 | Female | 54 | Proliferation |  | 4.8 |
| 10 | GNO_apop_rep2 |  |  | Apoptosis |  | 11.8 |
| 11 | GNO_prolif_rep3 | Female | 49 | Proliferation |  | 2.6 |
| 12 | GNO_apop_rep3 |  |  | Apoptosis |  | 6 |
| 13 | GCO_prolif_rep1 | Female | 80 | Proliferation | Gastric cancer | 5.2 |
| 14 | GCO_apop_rep1 |  |  | Apoptosis |  | 13.1 |
| 15 | GCO_prolif_rep2 | Male | 77 | Proliferation |  | 5.8 |
| 16 | GCO_apop_rep2 |  |  | Apoptosis |  | 7.8 |
| 17 | GCO_prolif_rep3 | Male | 56 | Proliferation |  | 5.4 |
| 18 | GCO_apop_rep3 |  |  | Apoptosis |  | 12.1 |
| 19 | Plasma | Male | 32 | NA | NA | 18.5 |


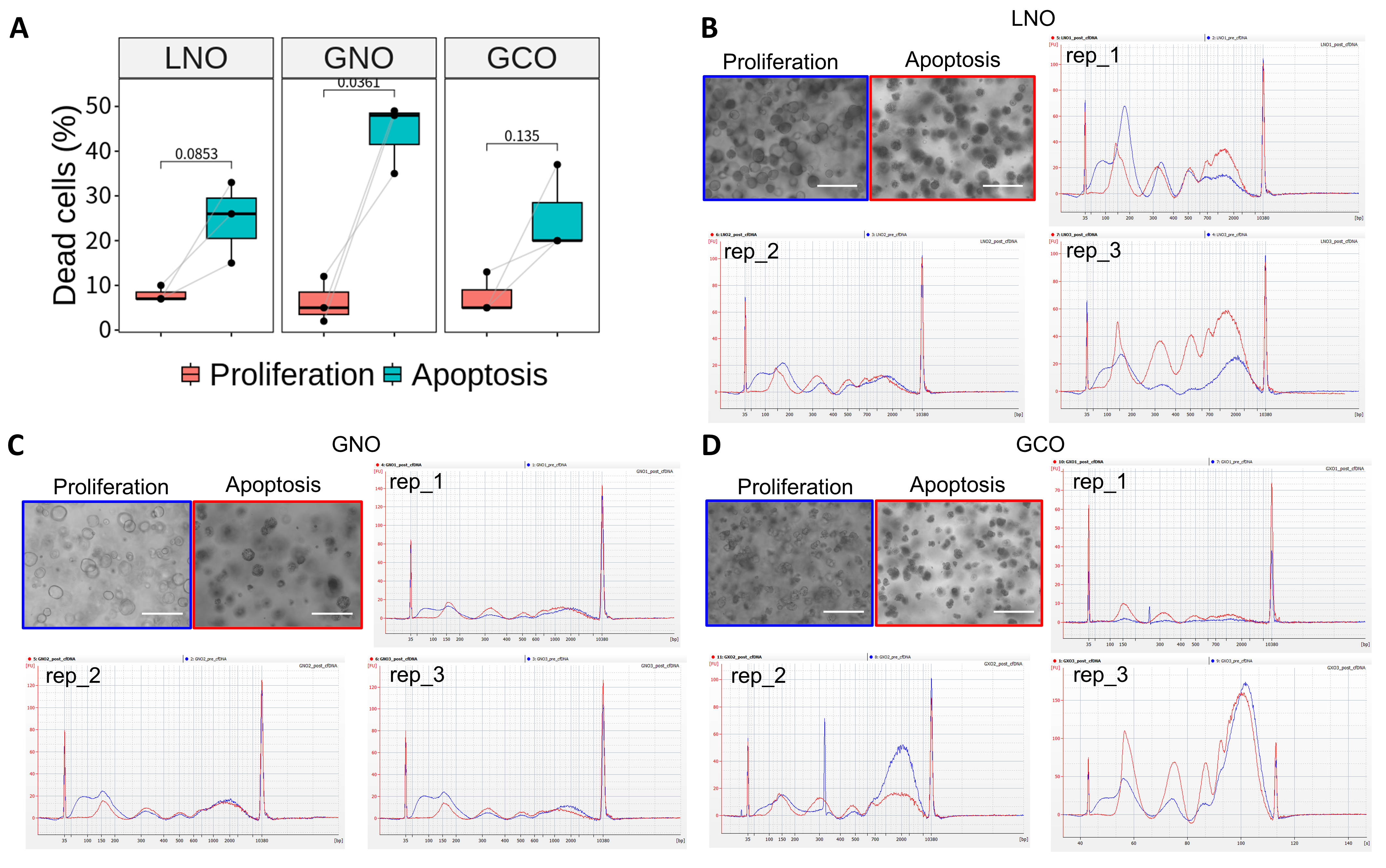


**Fig. S1.** **Comparison of the proliferation and apoptosis 3D organoid samples.** (A) The proportion of dead cells determined by AO/PI staining. (B–D) Microscopic morphological observations and capillary electrophoresis measurements of fragment sizes for the LNO (B), GNO (C), and GCO (D) samples. The blue lines indicate the proliferation samples and the red lines indicate the apoptosis samples. Representative microscopic images (×100) for one of the three replicates are shown. Electropherograms are presented for all three replicates. Scale bars, 1550 μm.


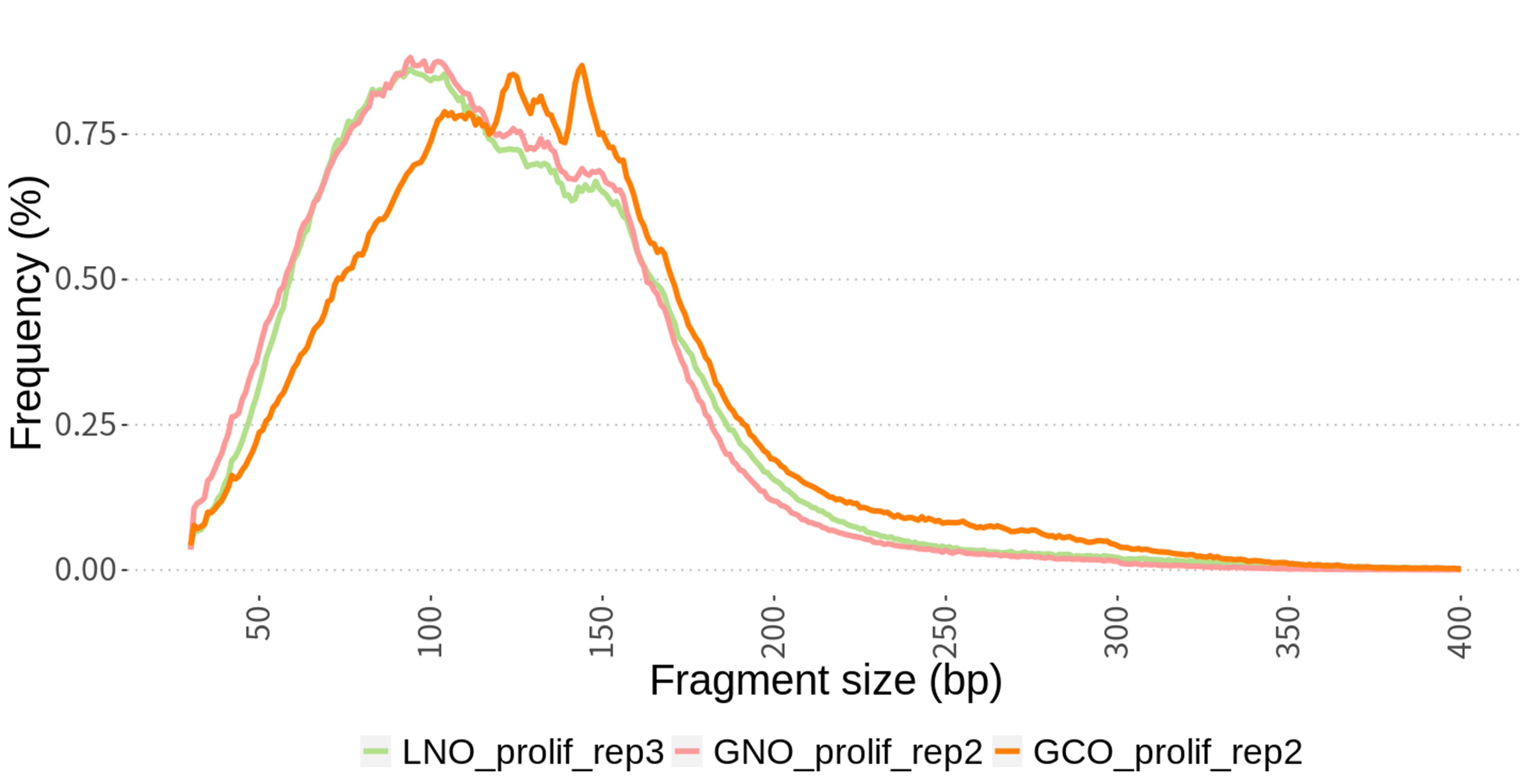


**Fig. S2.** **Distributions of the cfDNA fragment sizes sequenced using MiSeq.** For three proliferation organoid samples, fragment sizes were calculated based on read lengths for fragments less than 300 bp and insert size otherwise. Shallow sequencing (depth: 0.4×) was performed using Illumina MiSeq.


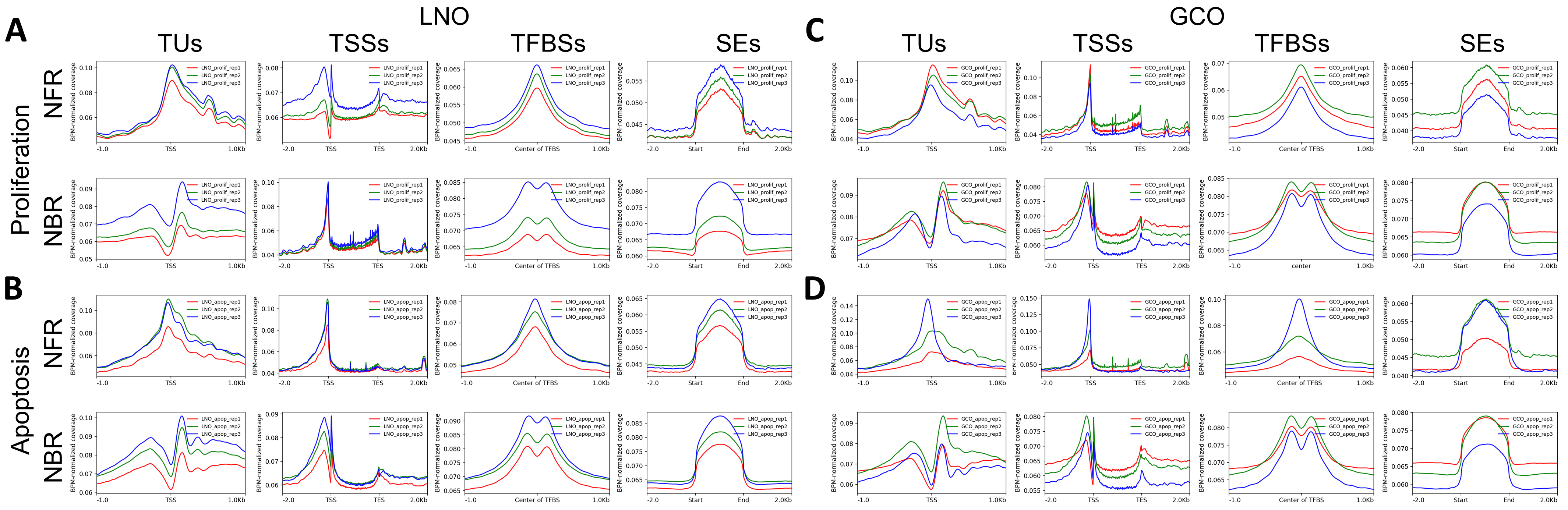


**Fig. S3.** **Footprints of DNA-binding proteins in cfDNA from organoid samples.** Bins per million mapped reads (BPM)-normalized depth around various protein binding regions, including transcription units (TUs), transcription start sites (TSSs) and end sites (TESs), transcription factor binding sites (TFBSs), and super enhancer regions (SEs), are shown for the proliferation (A and C) and apoptosis (B and D) LNO (A and B) and GCO (C and D) samples.


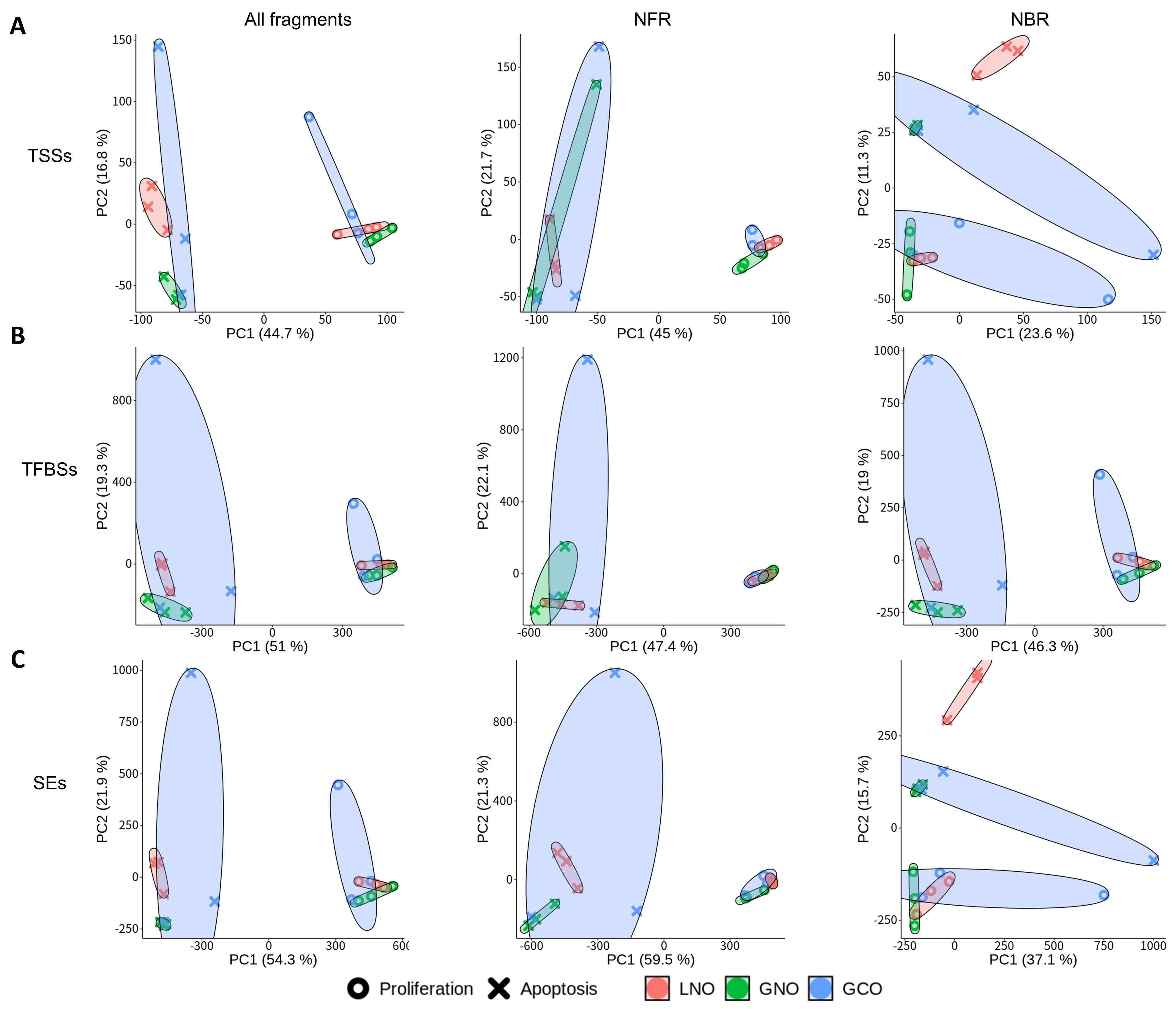


**Fig. S4.** **Principal component analysis using bins per million mapped reads (BPM)-normalized depths for regions of DNA-binding proteins.** Transcription start sites (TSSs) (A), transcription factor binding sites (TFBSs) (B), and super enhancer region (SEs) (C) for all fragments, NFR fragments, and NBR fragments. The shape of each datapoint indicates the state (proliferation or apoptosis) and the color indicates the type of organoid (LNO, GNO, or GCO). The ellipses encompass all samples of the same type and state.


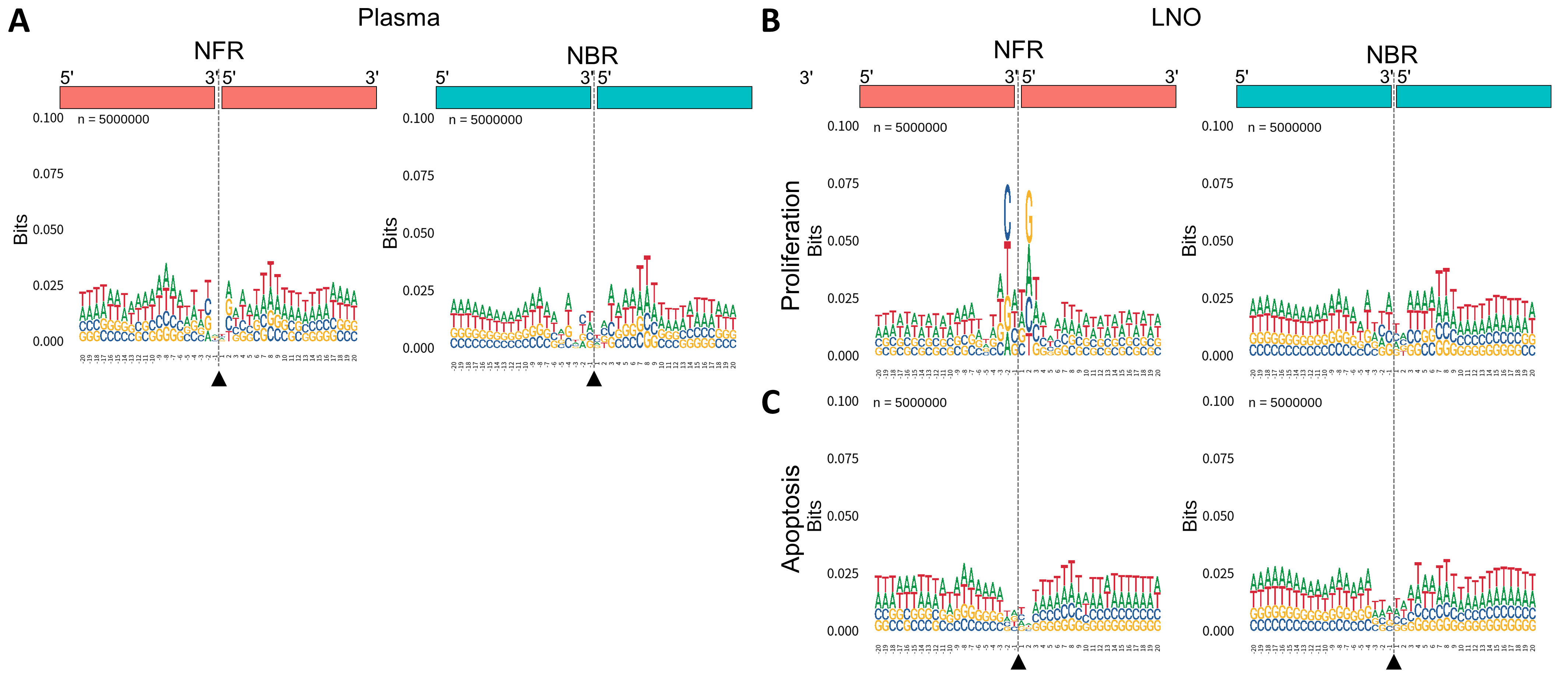


**Fig. S5.** **End motifs of cfDNA fragments.** (A) Results for the plasma sample. (B, C) Results for a proliferation LNO sample (B) and an apoptosis LNO sample (C), illustrating the different patterns between NFR and NBR fragments, as well as between proliferation and apoptosis samples. Breakpoints were defined as the points between the 3' and 5' ends and are indicated by gray dashed lines and triangle symbols.


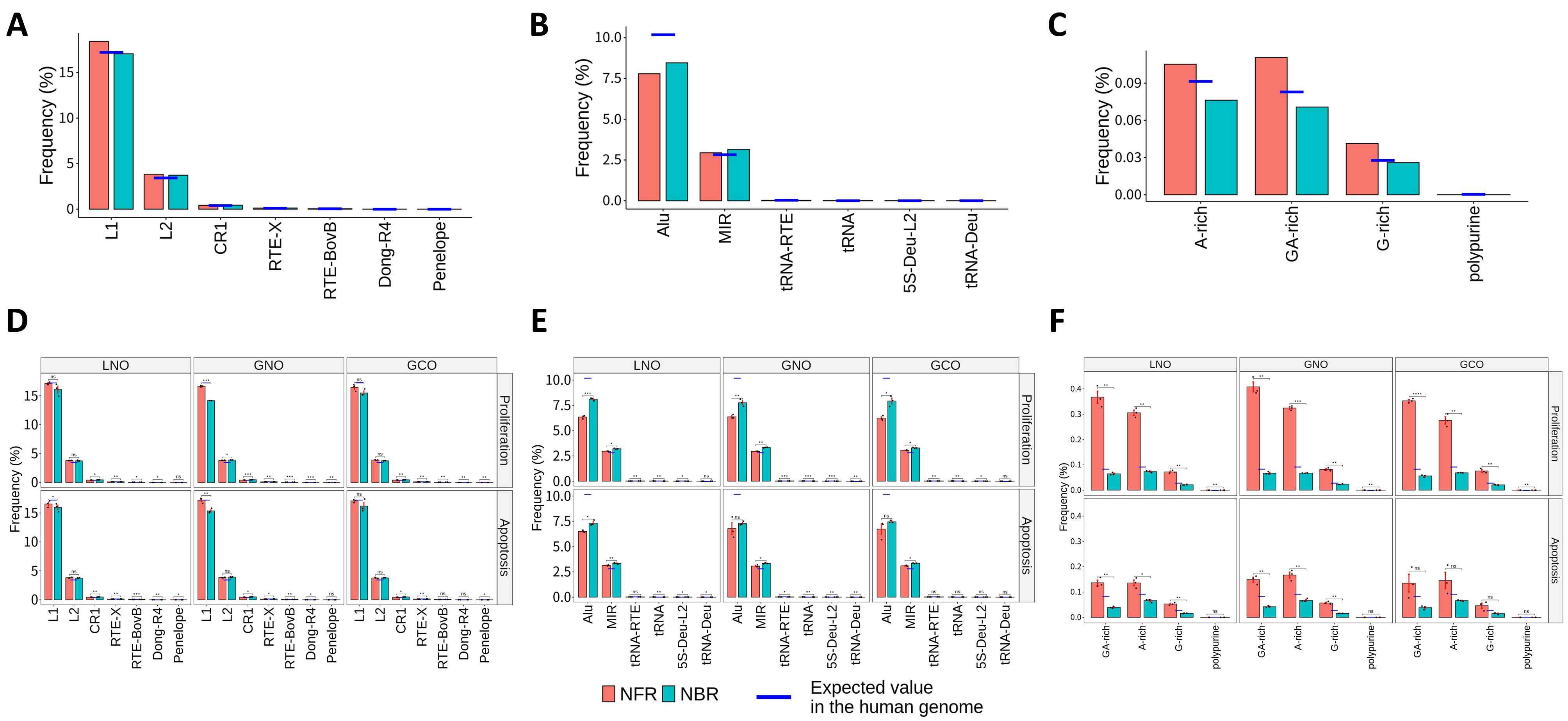


**Fig. S6.** **Proportions of the end positions of NFR and NBR fragments in repeat regions.** (A–C) The proportions of end positions of NFR and NBR fragments from the plasma sample in low-complexity repeats (A), LINEs (B), and SINEs (C). (D–F) The proportions of end positions of NFR and NBR fragments from the organoid samples in low-complexity repeats (D), LINEs (E), and SINEs (F). Black dots represent the value for each replicate. Red error bars represent the standard error of the mean. Statistical analyses were performed using the R package rstatix, with a Welch’s t-test (ns: not significant; ***p < 0.05, **p < 0.01, ***p < 0.001, and ****p < 0.0001).


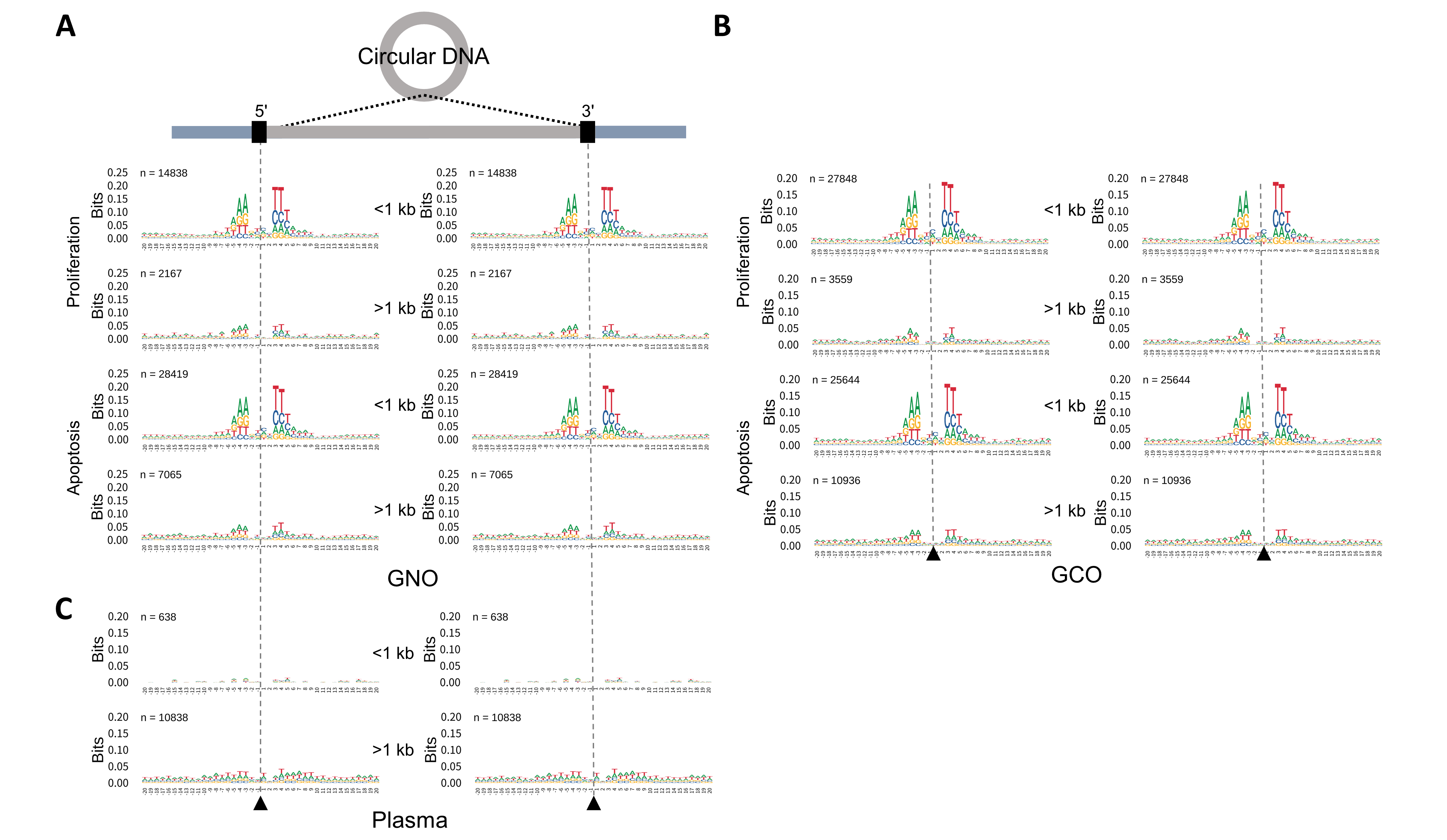


**Fig. S7.** **Analysis of DNA motifs surrounding circular DNA junctions.** Data are shown for the GNO (A), GCO (B), and plasma (C) samples. Data were similar for replicates of each organoid type and state. Junction breaks are indicated by gray dashed lines and triangles.


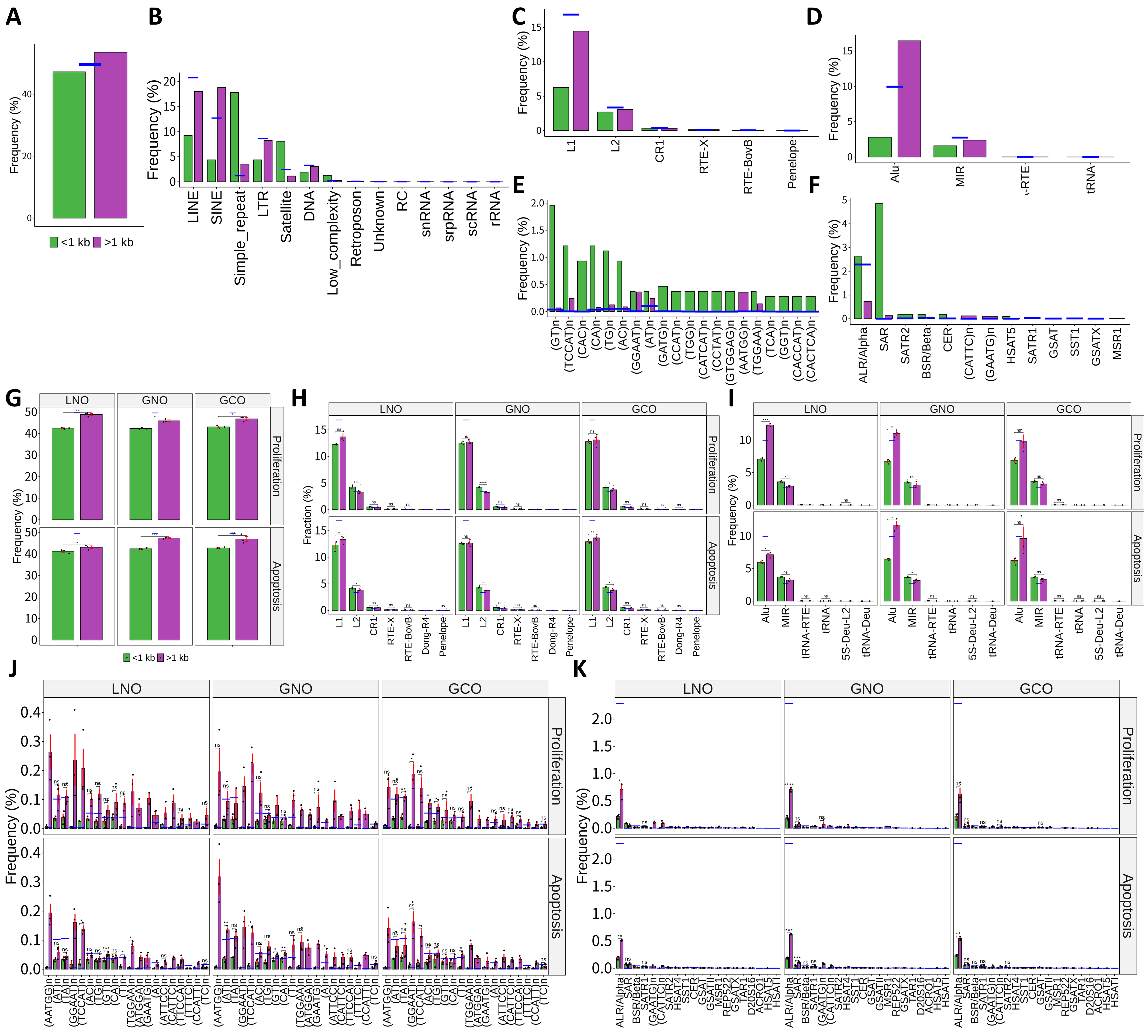


**Fig. S8.** **Distribution of junction breaks of circular DNA in repeat regions.** (A) The proportions of junction breaks of circular DNAs of the indicated lengths located in repeats for the plasma sample. (B–D) The proportions of junction breaks for the plasma sample in RepeatMasker-defined repeat classes (B), LINEs (C), SINEs (D), simple repeats (E), and satellites (F). (G) The proportions of junction breaks of circular DNAs of the indicated lengths located in repeats for the organoid samples. (H–J) The proportions of junction breaks for organoid samples in LINEs (H), SINEs (I), simple repeats (J), and satellite repeats (K).
